# Countering Triple Negative Breast Cancer via Impeding Wnt/β-Catenin Signaling, a Phytotherapeutic Approach

**DOI:** 10.3390/plants11172191

**Published:** 2022-08-24

**Authors:** Laleh Arzi, Homa Mollaei, Reyhane Hoshyar

**Affiliations:** 1 Department of Microbiology, Shahr-e-Qods Branch, Islamic Azad University, Tehran 37515-374, Iran; 2 Department of Biology, Faculty of Sciences, University of Birjand, Birjand 97175-615, Iran; 3 Cell biology Department, GenEdit, South San Francisco, CA 94080, USA

**Keywords:** herbal medicine, bioactive derivative, triple negative breast cancer, Wnt/β-catenin, anti-cancer

## Abstract

Triple negative breast cancer (TNBC) is characterized as a heterogeneous disease with severe malignancy and high mortality. Aberrant Wnt/β-catenin signaling is responsible for self-renewal and mammosphere generation, metastasis and resistance to apoptosis and chemotherapy in TNBC. Nonetheless, in the absence of a targeted therapy, chemotherapy is regarded as the exclusive treatment strategy for the treatment of TNBC. This review aims to provide an unprecedented overview of the plants and herbal derivatives which repress the progression of TNBC through prohibiting the Wnt/β-catenin pathway. Herbal medicine extracts and bioactive compounds (alkaloids, retinoids. flavonoids, terpenes, carotenoids and lignans) alone, in combination with each other and/or with chemotherapy agents could interrupt the various steps of Wnt/β-catenin signaling, i.e., WNT, FZD, LRP, GSK3β, Dsh, APC, β-catenin and TCF/LEF. These phytotherapy agents diminish proliferation, metastasis, breast cancer stem cell self-renewal and induce apoptosis in cell and animal models of TNBC through the down-expression of the downstream target genes of Wnt signaling. Some of the herbal derivatives simultaneously impede Wnt/β-catenin signaling and other overactive pathways in triple negative breast cancer, including: mTORC1; ER stress and SATB1 signaling. The herbal remedies and their bioactive ingredients perform essential roles in the treatment of the very fatal TNBC via repression of Wnt/β-catenin signaling.

## 1. Introduction

Breast cancer was reported as the most diagnosed cancer and the leading cause of cancer mortality worldwide among women in 2020 [1]. Advances in molecular techniques have prompted a conventional classification of breast cancer. The St. Gallen International Breast Cancer Conference 2011 allocated four molecular subtypes to breast cancers: Luminal A (ER+/PR+/HER2-/lowKi-67); Luminal B (ER+/PR+/HER2-/+/high Ki-67); HER2-overexpression (ER-/PR-/HER2+) and triple negative breast cancers (TNBCs) (ER-/PR-/HER2-) [2]. The TNBC is characterized as the most aggressive and fatal subtype, particularly in young women. It is famed as “the kiss of death”, being attributed with a poor chance of survival, early relapse, high proliferation and metastatic potential, heterogeneity and lack of efficient, approved targeted therapies [3,4,5]. Molecular analysis has evidenced that various signaling pathways are activated in the TNBC cells, such as Wnt/β-catenin, Hedgehog, Notch, TNF-α, Hippo and JAK-STAT [6]. However, since aberrant the Wnt/β-catenin pathway was reported as a predisposing factor of TNBC, prohibition of this pathway could be a worthwhile target for conquering TNBC progress [7,8,9]. The Wnt-signaling pathway is associated with cell proliferation, survival, invasion, metastasis and chemotherapy resistance in TNBC [10].

The Wnt signaling commences with the binding of Wnt ligands to the N-terminal extra-cellular cysteine-rich domain of the Frizzled (FZD) seven-pass transmembrane receptors and the LDL receptor-related proteins (LRP coreceptor). The trimetric complex (Wnt, FZD and LPR) recruits phosphorylated Disheveled (Dsh) and Axin, to prevent β-catenin phosphorylation. Therefore the APC/CK1/GSK-3β/Axin/β-catenin degradation complex is inactivated and the phosphorylation of β-catenin by GSK-3β is repressed. The accumulated β-catenin in the cytoplasm translocates into the nucleus, where it modulates the target gene expression with the T cell factor/lymphoid enhancer factor (Tcf/Lef) family of transcription factor [11]. Besides the classical canonical β-catenin-dependent mechanism of Wnt signaling, this pathway might progress through alternative signaling called “β-catenin independent mechanisms or non-canonical pathways” [12].

Although chemotherapeutic agents have been employed as exclusive treatments of TNBC, the resistance to chemotherapy agents (anthracyclines, taxanes, capecitabine, gemcitabine, eribulin), biomarker-based treatments and checkpoint-based immunotherapy has led to inconsequential responses to the medications. An extensive spectrum of clinical investigations has claimed the favorable influence of herbal remedies on the survival, immune enhancement and quality of life of cancer sufferers [13]. Moreover, due to their pharmacological safety, these compounds can be applied alone or complementary to chemotherapy in order to boost the therapeutic efficacy and diminish the chemotherapy-induced toxicity [14,15]. A myriad of investigations have verified the effectiveness of medicinal plant extracts and their bioactive components in targeting the Wnt pathway as novel therapeutic agents [16,17,18]. Accordingly, this paper is dedicated to the review and appraisal of plants and natural derivatives which suppress the progression of TNBC through prohibiting the Wnt/β-catenin pathway.

## 2. Natural Derivatives Targeting TNBC via Impeding the Wnt/β-Catenin Pathway

### 2.1. Saikosaponin D

Saikosaponin D, one of the main bioactive triterpenes, is extracted from the root of *Radix bupleuri* L. (Chaihu)—a known Chinese traditional medicine with broad applications. The Chinese Food and Drug Administration (CFDA) has approved fifteen clinical preparations of *Radix bupleuri* [19]. Saikosaponin D has been reported to suppress cancer cells through the downregulation of TNF-*α*-mediated NF-*κ*B, activation of autophagy or the blocking of the Wnt/β-catenin signaling pathway [20,21,22]. Wang et al. evinced the theory that Saikosaponin D reduced proliferation and activated apoptosis in various types of TNBC cell lines. The dynamic mass redistribution assay, TopFlash, and Western blot were applied to clarify the corresponding molecular mechanisms. They detailed that Saikosaponin D remarkably suppressed β-catenin and its downstream target genes (c-Myc and CyclinD1), leading to caspase-dependent cell death. The molecular docking of Saikosaponin D to the crystal structure of β-catenin suggests that it attaches to β-catenin via hydrogen bonds and hydrophobic interaction [23].

### 2.2. Echinacoside

Echinacoside is a phenylethanoid glycoside, isolated from the species of genus *Cistanches* (Orobanchaceae) and *Echinacea* (Asteraceae) [24]. Recent investigations have indicated the anti-proliferation, anti-migration and anti-invasion potencies of Echinacoside on TNBC cells (MDA-MB-231 and MDA-MB-468 cells), and illustrated that it exerted its effects via the inhibition of the Wnt/β-catenin signaling pathway; as the protein expression of crucial Wnt/β-catenin signaling factors (phosho-LRP6, total LRP6, phosho-Dvl2, active β-catenin, and total β-catenin) and the mRNA and protein expression levels of Wnt target genes (CD44, LEF1 and Cyclin D1) were reduced in the TNBC cell lines and the MDA-MB-231 xenograft mice model. The assessment of the murine tumor sizes and weights revealed the tumor-growth inhibitory activity of Echinacoside in the treated animals [25].

### 2.3. Sulforaphane

Sulforaphane, 4-methylsulfinylbutyl isothiocyanate, naturally originates from specific species of the *Brassica* vegetable family, most noteworthy, broccoli. The young sprouted broccoli seeds contain an immense amount of glucosinolate, which is metabolized by the myrosinase enzyme to Sulforaphane [26]. The potential of Sulforaphane to target breast cancer stem cells (BCSCs) in the cell and xenograft models of TNBC was evaluated. The sulforaphane treatment led to decreasing cell viability and induction of apoptosis in the SUM159 cells, via activation of caspase-3. The sulforaphane prevented mammosphere formation in the TNBC cells and remarkably reduced the Aldehyde Dehydrogenase (ALDH)-positive population cells in the cell and tumor models of TNBC. In addition, the tumor sizes in Sulforaphane-treated mice were lessened by 50% compared to the control mice. It should be mentioned that the Sulforaphane therapy had approximately no toxicity, as determined by measuring the mice body weight. Furthermore, the suppressive role of Sulforaphane on Wnt/β-catenin pathway was demonstrated, since β-catenin and Cyclin D1, the Wnt/β-catenin target genes, were down-expressed in SUM159 cells [27].

### 2.4. Gigantol

Gigantol, 4-[2-(3-hydroxy-5-methoxyphenyl) ethyl]-2-methoxyphenol, is a bibenzyl-type phenolic biomolecule extracted from various medicinal orchids, with numerous pharmaceutical benefits [28]. An investigation into the TNBC cell lines illustrated that Gigantol reduced viability and migration of the MDA-MB-231 and MDA-MB-468 cells, through impeding the Wnt/β-catenin signaling. The SuperTOPFlash assay reported that Gigantol prohibited the Wnt/β-catenin signaling via decreasing the level of phosphorylated LRP6, total LRP6 and cytosolic β-catenin, leading to a reduction in the expression of the Wnt target genes, Axin2 and Survivin [29].

### 2.5. Naringin

Naringin, a flavanone glycoside composed of the flavanone naringenin and neohesperidose, is a crucial bioactive ingredient of *Drynaria fortunei* (Kunze) J. Sm., *Citrus aurantium* L. and *Citrus medica* L. It occurs in citrus fruit and endows bitterness to the citrus juices [30]. The anti-tumor potential of Naringin on TNBC was assessed in both the cell (MDA-MB-231, MDA-MB-468 and BT-549 cells) and xenograft mice models. Western blot and immunohistochemistry assays demonstrated that Naringin elevated the p21 expression and diminished the Survivin and β-catenin levels. It was also found that the p21 and Survivin levels were regulated by β-catenin. The over-expression of β-catenin in the MDA-MB-231 and BT-549 cells remarkably reduced the repressive effect of Naringin on cell proliferation, whilst the knock-out of β-catenin caused the inhibition of TNBC cell growth. Shrinkage of the tumor was detected in the Naringin-treated mice. Therefore, it was supposed that Naringin could promote apoptosis (augmenting the activity of caspase 3) and G1 phase-arrest via hindering the Wnt/β-catenin pathway [31]. The nuclear β-catenin binds with and activates TCF4/LEF, which may switch on the transcription of p53 and c-Myc. C-Myc can elevate the expression of p14ARF, fas, Trail, fasR and DR4/5. FasR and DR4/5 promote the apoptotic extrinsic pathway, which is initiated by the binding of their respective ligands. This leads to the autoactivation of caspases-8 and -10, which sequentially boosts the catalytic activation of the effector caspase-3 [32].

### 2.6. Oxymatrine

Oxymatrine, a quinolizidine alkaloid compound isolated from the roots of *Sophoraflavescens Ait*, possesses diverse medicinal qualities [33]. Oxymatrine reinforced the anti-tumor activity of Bevacizumab, by restraining invasion and metastasis induced by Bevacizumab in TNBC cells, via inactivating the Wnt/β-Catenin pathway [34]. The monoclonal antibody, Bevacizumab, is applied, in combination with neoadjuvant chemotherapy, as the first-line treatment for metastatic breast cancer. It demonstrated effective anti-angiogenesis potential, whilst also augmenting the metastatic tendency of the TNBC cells [35,36]. Xie et al. showed that Oxymatrine inhibited the migration and invasion via reverting the EMT phenotype. In the treated group, the expression level of the mesenchymal-associated genes, N-Cad and Vim, and the EMT-related transcription factors including ZEB, Snail and Slug, were elevated, whereas the expression of the epithelial-associated gene E-Cad declined in comparison to the control. Oxymatrine decreased the subpopulation of the TNBC stem-like cells through repressing the Wnt/β-Catenin pathway, since the expressions of β-Catenin and its downstream oncoproteins were attenuated. In addition, Oxymatrine reduced the tumor growth in tumor-bearing mice and reduced the risk of relapse and metastasis by abating the self-renewal capacity of the cancer stem cells (CSCs). It is worth noting that Oxymatrine synergistically amplified the anti-angiogenic potency of Bevacizumab. This alkaloid suppressed the cell viability of HUVEC cells dose-dependently and reduced the formation of tumor neovascularization in the combination-treated mice [34].

### 2.7. Silibinin

Silibinin, an essential bioactive flavonolignan extracted from milk thistle (*SilybumMarianum* L. Gaertn), elucidated chemopreventive and chemo-sensitizing properties against various cancers [37]. It was demonstrated that Silibinin abated the proliferation of TNBC cells and suppressed the phosphorylation and expression of endogenous LRP6 in the MDA-MB-231 cells [38]. Growing evidence indicated that the LRP6 is a pivotal Wnt co-receptor, valued in therapeutic strategies for TNBC [39]. Therefore, it was speculated that Silibinin, as an inhibitor of Wnt/β-catenin signaling, could be considered as a natural propitious anti-tumor remedy.

### 2.8. Rottlerin

Rottlerin, a polyphenol extracted from the Asian Kamala plant *Mallotus philippinensis*, is regarded as a multifaceted anti-cancer natural agent in various cancers [40]. Lu’s research group surveyed the molecular mechanism of the anti-proliferative potency of this polyphenol on various prostate and breast cancers, including the TNBC cell line (MDA-MB-231). Rottlerin impaired the Wnt/β-catenin signaling, since it diminished the expression of the cytosolic-free human β-catenin, total cellular human β-catenin, human LRP6, phospho-LRP6 and axin2. Subsequently, it was illustrated that Rottlerin-mediated LRP6 downregulation was unassociated with AMP-activated protein kinase (AMPK). Furthermore, Rottlerin disrupted the mTORC1 signaling, as it remarkably repressed P70-S6K, phospho-P70-S6K, S6 and phospho-S6in TNBC cells. The Rottlerin lowered the expression of the common target oncogenes of the Wnt/β-catenin- and mTORC1-signaling pathways, Cyclin D1 and Survivin [41]. Mounting evidence supported the crosstalk between the Wnt/β-catenin and the mTORC1 signaling, the activation of the Wnt/β-catenin-signaling upregulates the mTORC1 signaling in the cancer cells [42]. Hence, this investigation assumed that the anti-cancer potential of Rottlerin was associated with the dual suppression of the Wnt/β-catenin and mTORC1 signaling.

### 2.9. Baicalin

Baicalein is the flavonoid glucoside of *Scutellaria baicalensis* Georgi, Lamiaceae, which has anticancer potential. This medicinal plant is native to China, Korea, Mongolia and in the Russian Far East and Siberia [43]. The anti-metastatic potency of Baicalin and its underlying mechanisms have been evaluated in the most aggressive type of breast cancer. Baicalin attenuated the survival, migration and invasion of TNBC cells (MDA-MB-231 and 4T1), whereas it illustrated no noticeable effect on the proliferation of the cancer cells. Moreover, Baicalin-treated tumor-bearing mice possessed less liver and lung metastatic lesions compared to the control mice. In addition, it was elucidated that this flavonoid functioned as an anti-metastatic agent via inverting EMT and downregulating the expression of β-catenin in the cell and mice models of TNBC. These findings recommend Baicalin in conjunction with conventional chemotherapy agents in the treatment of TNBC patients [44].

### 2.10. Baicalein

Baicalein is a crucial bioactive flavonoid found in the dried root of *Scutellaria baicalensis* Georgi, a traditional Chinese medicine belonging to the *Lamiaceae* family. Extensive research displayed that Baicalein activated apoptosis, induced cell cycle arrest and suppressed metastasis in different cancers [45]. A survey assessed the effect of Baicalein on the cell and animal models of TNBC. They confirmed that Baicalein decreased the viability, migration and invasion of the MDA-MB-231 cells dose- and time-dependently. In accordance with the cell assays’ findings, it was revealed that Baicalein gavage in mice reduced the metastasis rate in the liver and lung tissues of the prevention and therapy group compared to the control mice. In addition, in the Baicalein-treated MDA-MB-231 cells and mice, the EMT process was inhibited, since the expression of E-Cad (molecular marker of epithelial cells) was elevated, while Vim (molecular marker of mesenchymal cells) and Snail (transcription factor) expressions diminished. In addition, Baicalein exposure promoted the downregulation of Wnt1, β-catenin and AT-rich sequence-binding protein-1 (SATB1) in the MDA-MB-231 cells and tumor-bearing mice. It should be mentioned that Baicalein reduced the expression of Cyclin D1 and Axin2 in TNBC cells. It can be deduced that Baicalein restricted metastasis in the cell and animal models by reverting EMT, which may be associated with impeding SATB1 and the Wnt pathway [46].

### 2.11. Epigallocatechin Gallate

Epigallocatechin gallate, a biopolyphenol, is the most abundant and influential antioxidant extracted from green tea [47]. Hong et al.’s investigation into breast cancer patients (females suffering from invasive ductal carcinoma, having undergone a curative operation) illustrated that β-catenin is upregulated in breast cancer tissue compared with its expression in normal adjacent tissues, and that the β-catenin overexpression is correlated with lymph node involvement, high tumor stage and ER-negative status. They also showed that Epigallocatechin gallate treatment reduced the cell viability and expressions of β-catenin, P-AKT and Cyclin D1 in the MDA-MB-231 cells, and that pretreatment with phosphatidylinositol-3 (PI3) kinase inhibitors (25 µm LY294002 or 5 µM Wortmannin) elevated the suppressive effect of Epigallocatechin gallate on the β-catenin expression. These results imply that Epigallocatechin gallate’s cytotoxic potential on TNBC is related to the inactivation of Wnt/β-catenin [48]. This finding is in accord with a previous survey illustrating that β-catenin is able to attach to PI3 kinase, which may play a role in the stabilization of β-catenin. Therefore, the repression of PI3 kinase may lead to the declined expression of β-catenin [49].

Supporting findings were obtained by Kim’s research group. They displayed that Epigallocatechin gallate obstructed growth, migration and invasion. This polyphenolic compound blocked Wnt signaling and its target gene, c-Myc in MDA-MB-231. They previously cited the HMG-Box Transcription Factor 1 (HBP1) as a negative transcriptional regulator of Wnt signaling [50]. HBP1 is a high mobility group (HMG) box transcription factor, similar to LEF and TCF in the Wnt pathway [51]. Therefore, they evaluated the capability of Epigallocatechin gallate in targeting the Wnt signaling through HBP1. They proposed that the anti-proliferative and anti-metastatic properties of Epigallocatechin gallate emerged from the suppression of the Wnt signaling via targeting the HBP1 transcriptional repressor [50].

### 2.12. Cardamonin

Cardamonin, (2,4-dihydroxy-6-methoxychalcone) is a naturally occurring chalcone, commonly derived from different plants of the *Zingiberaceae* family. There is mounting evidence supporting the multi-potency anti-cancer activities of Cardamonin via modulating various signaling pathways, transcriptional factors, cytokines and enzymes, such as mTOR, NF-κB, Akt, STAT3, Wnt/β-catenin and COX-2 [52]. The breast tumor-suppressive role of Cardamonin and its underlying molecular mechanism was assessed in cell and mice models of TNBC. The MTT assay revealed the selective cytotoxicity of Cardamonin on TNBC cells, without influencing the normal breast epithelial cells, MCF-10A. The Cardamonin activated the mitochondrial pathway of apoptosis through modulation of Bax, Bcl-2, Cyt-C, caspase-3 and PARP in MDA-MB-231 and BT-549 cells. Cardamonin impaired the migration and invasion of TNBC cells via impeding EMT. Furthermore, the assays have illuminated the role of Cardamonin reduction in the stability and nuclear translocation of β-catenin via activating GSK3b. Measuring the tumor volumes demonstrated that the tumor sizes were reduced in the murine model of TNBC [53].

### 2.13. Inotodiol

Inotodiol is a natural lanostane-type triterpenoid isolated from *Inonotus obliquus* (*Chaga mushroom*), an edible fungus which inhabits birch trees [54]. A previous piece of research on the cell and mice models of colorectal cancer claimed that the aqueous extract of *Inonotus obliquus* exhibited potent anti-inflammatory and anti-proliferative properties through the downregulation of Wnt/β-catenin [55]. The effect of Inotodiol was assessed on the progress of breast cancer in 7, 12-dimethylbenz(a)anthracene (DMBA)-administered diabetic rats [56]. It was demonstrated that the DMBA induced breast cancer in mice through the activation of Wnt/β-catenin signaling [57]. The results indicated that Inotodiol lowered blood glucose (fasting blood glucose levels and oral glucose tolerance test) and plasma levels of cholesterol, triglyceride and HDL, and elevated the antioxidant enzymes activities in Sprague-Dawley (SD) rats. It was observed that in the Inotodiol-treated rats, the expression of the proliferating cell nuclear antigen (PCNA), a tumor proliferation marker, decreased. Subsequently, the deregulation of β-catenin and its downstream target genes (c-Myc and Cyclin D1) and the induction of apoptosis (Caspase-3 and PARP gene expressions were strikingly upregulated) eventuated. It was concluded that Inotodiol regulated the blood glucose in diabetic rats and subsequently repressed tumor progression in breast cancer by activating apoptosis via the downregulation of the Wnt/β-catenin pathways [56]. This finding is in accord with research that established Wnt/β-catenin as a linkage between high glucose and cancer [58].

### 2.14. Schisandrin A

Schisandrin A is the most dominant lignan (polyphenolic compounds) present in the fruit of *Schisandra chinensis* (Turcz.) Baill. [59]. The anti-tumor efficacy of Schisandrin on TNBC cells has been studied. It has been demonstrated that Schisandrin (25, 50 and 100 µM) reduces viability, inhibits colony formation, induces nuclear fragmentation, condenses chromatin, arrests the cell cycle in G1 phase and promotes apoptosis by elevating Bax and P53 and reducing the Bcl2 proteins in TNBC cell lines. The oral gavages of Schisandrin (25 mg/kg) induced no apparent toxicity in the organs of female BALB/c nude mice and the animals showed no appreciable changes in body weight and possessed smaller tumors. Following Schisandrin administration, an increase in the endoplasmic reticulum stress-specific protein, ATF4, CHOP and phosphorylated elf2α, and decrease in the Wnt signaling-associated proteins, β-catenin and phosphorylated GSK3β, were detected in the MDA-MB-231 and BT-549 cells and tumor tissues [60]. The accumulation of extra ER stress enhanced cancer cell death and the ER stress activators were assumed as the potential candidates for the remedy of TNBC [61]. Therefore, it can be concluded that Schisandrin exerts its anti-tumorigenic effects through the regulation of the Wnt/ER stress signaling pathway.

### 2.15. Resveratrol

Resveratrol (3,4’, 5-trihydroxy-trans-stilbene) is a plant polyphenolic derivative, widely found in grapes, berries and peanuts [62]. The clinical finding of CSCs in breast cancers have displayed a relation between the fraction of CSCs and poor prognosis [63]. Accordingly, Fu et al. assessed the repressive potential of Resveratrol on BCSCs. They found that Resveratrol inhibited the proliferation of SUM159. It diminished the augmented ALDH-positive breast cancer cells and reduced the number and size of mammospheres. The injection of Resveratrol (100 mg/kg) to female non-obese diabetic/severe combined immunodeficiency (NOD/SCID) tumor-burden mice reduced the tumor volume and ALDH population in tumor cells. The induction of autophagy by resveratrol was displayed in the BCSCs from the SUM159 cells, since the number of autophagosomes and the expression of Lc3-II, Beclin1 and Atg7 genes required for autophagosome formation were elevated. The Western blot results illustrated that Resveratrol attenuated the expression of β-catenin and Cyclin D1 in BCSCs and xenograft mice. The overexpression of β-catenin by transected plasmid of pcDNA3-S33Y β-catenin or repression of autophagy by chloroquine eliminated the suppressive effect of Resveratrol on the Wnt/β-catenin signaling [64]. The Wnt/β-catenin-signaling pathway is pivotal in the management of BCSCs self-renewal and autophagy [65], therefore it was inferred that Resveratrol suppressed the BCSCs and activated autophagy via impeding the Wnt/β-catenin-signaling pathway, while exhibiting no cytotoxicity on the noncancerous cells and the treated mice [64].

### 2.16. Deguelin

Deguelin is one of the major naturally occurring rotenoids, extracted from *Mundulea sericea* L., belonging to the *Leguminosae* family [66,67]. It was observed that Deguelin suppressed the growth of the MDA-MB-231 cells dose-dependently, while no significant growth restriction was reported for up to one week in the MCF-12F cells (normal immortalized human mammary epithelial cells). The Deguelin treatment prompted cell cycle arrest at the S phase and induced apoptosis in the TNBC cells. The microarray analysis indicated that Deguelin upregulated the Wnt/β-catenin inhibitors (WIF-1, DDK4) and some of the cadherin family members (CDH3, CDH7, CDH9), while downregulating the Wnt/β-catenin activators (Wnt14, Wnt2B, Wnt3) and Snail. The Western blot results demonstrated that following Deguelin exposure, no appreciable changes were observed in GSK-3β(repressor of β-catenin), in spite of the decreased expression of p-GSK-3β (inactive form of GSK-3β), β-catenin and Cyclin D1.Therefore, it was concluded that Deguelin exerted its therapeutic benefits on TNBC via inhibition of Wnt/β-catenin by activation of GSK-3β, and subsequently β-catenin destruction [67].

### 2.17. Hydroxytyrosol

Hydroxytyrosol (3,4-dihydroxyphenylethanol) is a phenolic alcohol extracted from olive oil [68]. Cruz-Lozano et al.’s investigation into triple negative breast cancer cell lines displayed that hydroxytyrosol diminished the mammosphere-formation efficiency (MSFE) and decreased the volume of the second generation of mammospheres (assembled from the detached primary mammospheres), thus reducing BCSC self-renewal. This herbal phenol decreased ALDH^+^ and mesenchymal–like CD44^+^/CD24^−/low^ BSCS subpopulations. In addition, it suppressed the migration and invasion of various TNBC cell lines (SUM159PT, BT549, MDA-MB-231, and Hs578T) [69]. These results are in line with the role of cells with high CD44 and low CD24 expressions and high ALDH activity in vast incidences of metastasis, therapy resistance and tumor relapse in breast cancers [70]. Hydroxytyrosol reduced the expression of the EMT-related transcription factors (Zeb, Slug and Snail) and the mesenchymal marker (Vim), and increased the epithelial marker (ZO-1), as well as downregulating the Wnt/β-catenin proteins (p-LRP6, LRP6, β-catenin, and Cyclin D1). Hence, Hydroxytyrosol, as a chemopreventive agent, reduced BCSCs and the metastatic potential of TNBC via disturbing EMT and the Wnt/β-catenin pathway [69].

### 2.18. Fucoidan

Fucoidan, a polysaccharide obtained from brown seaweed and some marine invertebrates, consists of considerable amounts of L-fucose and sulfate ester groups. It may also contain other monosaccharides (mannose, galactose, glucose, xylose) and uronic acids, acetyl, sulfate and protein [71]. It was reported that Fucoidan (50, 100, and 200 µg/mL) inhibited 4T1 cell growth, induced apoptosis in the 4T1 cells and the tumor tissue of BALB/c mice (G1 arrest in cell cycle was detected) and inhibited the TCF/LEF reporter activity dose-dependently. The Western blot analysis showed that fucoidan treatment downregulated the expression of β-catenin and its downstream target genes, c-Myc, Cyclin D1 and Survivin, in 4T1 and tumor-bearing mice. The immunohistochemical staining confirmed the Western blot results, as Fucoidan attenuated the β-catenin-positive cells [72]. These results are consistent with the evidence that the downregulation of the β-catenin proteins prohibits the TCF/LEF reporter activity [73]. Therefore, it can be inferred that the anti-proliferative and apoptotic potency of Fucoidan is associated with restraining the Wnt/β-catenin signaling pathway.

### 2.19. Jatrophone

Jatrophone is a macrocyclic diterpene, consisting of an oxaspiro core and several electrophilic centers. Jatrophone is isolated from the *Euphorbiaceae* family, such as *Jatropha isabelli* and *Jatropha gossypiifolia* [74]. Fatima’s research group determined the cytotoxicity of Jatrophone on different subtypes of TNBC: mesenchymal stem-like (MDA-MB-231 and MDA-MB-157), basal-like-1(HCC-38 and MDA-MB-468) and patient-derived xenograft. They observed that it arrested the cell cycle in the S phase even more efficiently than the classic Wnt inhibitor, ICG-001, and induced late apoptosis in MDA-MB-231. The Topflash reporter and immunofluorescence outcomes demonstrated that Jatrophone impeded the Wnt/β-catenin signaling between the receptor complex and β-catenin (early level of Wnt/β-catenin pathway). The qPCR and immunoblot assays revealed that Jatrophone reduced the expression of Wnt/β-catenin direct target genes (reducing the mRNA level of Birc5, Axin2, Hmga2, Myc, PCNA and Ccnd1 and lowering the protein level of Axin2, Hmga2, Myc, PCNA, CyclinD1 and activated β-catenin). Likewise it decreased the migration of MDA-MB-231cells by downregulation of EMT markers, such as Slug, fibronectin and Vim. Hence, the anti-proliferative and anti-migration potency of Jatrophoneis applied through inhibiting the Wnt/β-catenin pathway by the elimination of nuclear-activated β-catenin and the downregulation of the downstream Wnt target genes [75].

### 2.20. Luteolin

Luteolin, 3,4,5,7-tetrahydroxy flavone, is a natural flavonoid, extensively present in many fruits and vegetables, such as celery, sweet bell peppers, carrots, broccoli, onion leaves and parsley [76]. The anti-metastatic potential of Luteolin on MDA-MB-231 and BT5-49 were evinced by wound healing and Transwell chamber assays. Luteolin caused an alteration in the morphology of TNBC cells from mesenchymal to oval epithelial. Immunofluorescent staining, Western blot and qPCR results confirmed the reversion of the EMT phenotype, i.e., the mesenchymal markers (N-Cad and Vim), and the EMT related transcription factors (Snail and Slug) were downregulated, while the epithelial markers (E-Cad and claudin) were upregulated, and the down-expression of β-catenin in the TNBC cells and mice tumor tissues was observed. The mice model assays exhibited that the Luteolin-treated tumor-burden mice possessed less metastatic colonies in the lungs. Thus, the anti-metastatic behavior of Luteolin on TNBC stemmed from its ability to reverse EMT through the downregulation of β-catenin [77].

### 2.21. Triptolide

Triptolide, diterpene triepoxide, a natural component, is extracted from the traditional Chinese medicinal plant *Tripterygium wilfordii* Hook F. [78]. It was observed that triptolide (10, 25 and 50 nM) lowers cell proliferation and increases the apoptosis rate in the MDA-MB-231 cells dose-dependently. Triptolide treatment downregulated the expression level of β-catenin. The therapeutic efficacy of Triptolide (anti-proliferative and apoptotic potency) on TNBC cells was applied via interfering with the Wnt/β-catenin signaling [79].

### 2.22. Astragalus Polysaccharide

Astragalus polysaccharide, a water-soluble hetero polysaccharide, consisting of α-1,4-(1,6)-glucan, rhamnus-galacturonic acid polysaccharide I, arabinogalactan polysaccharide and arabinogalactan protein polysaccharide. It is an essential bioingredient isolated from the roots of *Astragalus membranaceus* L., a traditional Chinese remedy [80,81]. The anti-proliferative property of *Astragalus* polysaccharide on human triple negative breast cancer cells (MDA-MB-231) was evaluated and evidenced by MTT and Ki67 immunofluorescence staining assays. Furthermore, it was found that Astragalus polysaccharide therapy prohibited the migration and invasion of these cells. These findings were consistent with RT-qPCR, Western blot and immunofluorescence staining analysis, as they confirmed the impediment of EMT via the downregulation of the mesenchymal marker Vim and the EMT-related transcription factor Snail, and the upregulation of E-Cad. Following Astragalus polysaccharide exposure, the expression of β-catenin and its downstream oncoproteins c-Myc and Cyclin D1 were reduced. The repressive potential of this hetero-polysaccharide on metastasis was exerted via altering the expression of the Wnt/β-catenin- and EMT-related genes. Moreover, it was demonstrated that lithium chloride (LiCl), an agonist of the Wnt/β-catenin pathway, inverted the suppressive effect of Astragalus polysaccharide on the Wnt/β-catenin pathway [81].

### 2.23. Quercetin

Quercetin (3,3’,4’,5,7-pentahydroxyflavone) is a yellow pigment bioflavonoid contained in numerous fruits, vegetables, seeds, nuts, green tea and red wine [82,83]. Srinivasan’s group investigated the effect of this flavonoid on human triple negative breast cancer cells and showed that quercetin reduced the survival rate, proliferation, migration and invasion of the MDA-MB-231 and MDA-MB-468 cells. Co-treatment of Doxorubicin amplified the anti-migration potency of Quercetin. It induced a phenotype transformation in human TNBC cells from a fibroblast spindle-like form to a cobblestone epithelial shape. Accordingly, Vim protein expression was reduced and E-Cad protein and mRNA expressions were enhanced. The Quercetin exposure of MDA-MB-231 localized the β-catenin in the cytoplasm and reduced the expressions of Cyclin D1, C-Myc and P-AKT [83]. It is reported that the abnormal aggregation and nuclear localization of β-catenin is related to tumorigenic activation [84]. It could be assumed that the anti-proliferative and anti-metastatic properties of Quercetin results from inhibiting Wnt/β-catenin signaling via impeding the presence of β-catenin in the nucleus and downregulation of its downstream target genes.

### 2.24. Quinacrine

Quinacrine (Atabrine, Mepacrine, 4-*N*-(6-chloro-2-methoxyacridin-9-yl)-1-*N*,1-*N*-diethylpentane-1, 4-diamine), is a quinine derivative extracted from the bark of the cinchona tree [85]. Preet et al.’s survey on human TNBC cells demonstrated that Quinacrine attenuated the activity of the Wnt transcription factor TCF/LEF, diminished the expressions of β-catenin and Cyclin D1 and elevated the expression of adenomatous polyposis coli (APC). It reduced survival and proliferation, and induced DNA damage and apoptosis in the MDA-MB-231 cells. Moreover, it was found that the knock-down of APC by siRNA might block the effects of Quinacrine, suggesting a correlation between Quinacrine function and APC. It was proved that Quinacrine inhibited the topoisomerase activity. The research team demonstrated that Quinacrine elevated the APC expression via suppression of topoisomerase activity, eventually repressing WNT/TCF singling in TNBC [86,87].

Lycopene, the main carotenoid in tomatoes, synergistically reinforced the anti-proliferative capability of Quinacrine. It is worth noting that lycopene, quinacrine or their combination possessed considerably less toxicity on MCF-10A (normal epithelial cells). The treatment of cells with Quinacrine followed by Lycopene, decreased the TCF/LEF activity compared to only Quinacrine therapy. The co-treatment with Quinacrine and Lycopene upregulated the APC expression while it downregulated β-catenin and Cyclin D1. Lycopene improved the anti-proliferation potential of Quinacrine by prohibiting the WNT/TCF signaling in the MDA-MB-231 cells [87].

### 2.25. Curcumin

Curcumin (1,7-bis (4-hydroxy-3-methoxyphenyl)-1,6-heptadiene-3,5-dione) is a yellow-colored natural hydrophobic polyphenol occurring in the rhizome of the *Curcuma longa* L. [88]. Prasad’s research team examined the growth repressive activity of curcumin on the MDA-MB-231 cells. They exhibited that Curcumin inhibited the proliferation and promoted apoptosis by arresting cell cycle at the G2/M phase in human TNBC cells. In addition, the Curcumin therapy attenuated the protein expression of the Wnt/β-catenin components Disheveled, β-catenin, Cyclin D1 and Slug, with negligible modification in the GSK3β and E-Cad levels after 12 h. The immunofluorescence analysis supported the Western blot results, since following the Curcumin treatment of the MDA-MB-231 cells, a decrease in the cytoplasmic and nuclear expressions of Disheveled and a reduction in the nuclear expression of β-catenin, Cyclin D1 and Slug were reported [89].

In another piece of research on SUM159 cells, it was displayed that the Curcumin treatment reduced the tumor sphere formation and downregulated the BCSCS markers (CD44, ALDH1A1, Nanog and Oct4), cell-proliferation proteins (PCNA and Cyclin D1) and the anti-apoptotic protein (Bcl2), and upregulated the apoptotic proteins (Bax, Caspase8, Caspase9 and cleaved Caspase3) [90]. It is worth noting that β-catenin activates TCF4/LEF, which may promote the transcription of p53 and c-Myc. C-Myc overexpressed p14ARF, fas, Trail, fasR and DR4/5. FasR and DR4/5 initiate the apoptotic extrinsic pathway by the attaching of their respective ligands. This leads to the autoactivation of caspases-8 and -10, which in turn stimulate the catalytic activation of the effector caspase-3. Another goal of caspase-8 is the pro-apoptotic protein Bid, which is hydrolyzed to tBid, inducing Bax oligomerization and mitochondrial depolarization with a release of cyt c. In addition to the activation of caspase-9, these events amplify the apoptotic pathway [32]. Curcumin repressed Wnt/β-catenin through the downregulation of p-GSK3β, β-catenin and c-Myc. The effect of Curcumin on Wnt/β-catenin was assessed by applying LiCl, which activated the Wnt/β-catenin pathway by blocking GSK3β. It reduced the restrictive effects of Curcumin on tumor sphere formation and the expression of the BCSCs markers. Furthermore, the upregulation of β-catenin in the SUM159 cells by transfection with the control vector and β-catenin plasmids abrogated the Curcumin-induced CD44 and Nanog down-expression [90]. Consistent with the above findings, a survey on normal breast tissue reported that Curcumin could serve as a potent inhibitor of breast cancer. It prohibited stem cell self-renewal through the suppression of Wnt signaling. The deregulation and acquisition of self-renewal potency in stem cells may interfere with carcinogenesis [91].

### 2.26. Crocin

Crocin, 8, 8-diapocarotene-8, 8-dioic acid, is the essential water-soluble carotenoid extracted from the dried stigmas of *Crocus sativus* L. (saffron) [92,93]. Arzi et al. surveyed the anti-metastatic effect of Crocin on 4T1 cells, establishing the anti-proliferative, anti-migration and anti-invasion properties of Crocin on TNBC cells. In another study, our team demonstrated that the Crocin-treated tumor-burden mice had more body weight, higher survival chances, smaller tumor sizes and less metastatic colonies in their livers and lungs compared to the control animals. It should be noted that Crocin generated no toxicity on BALB/c mice, since no apparent alteration in biochemical markers and body weight was observed. In addition, this carotenoid was reported to repress Wnt signaling via the down-expression of Wnt target genes, Fzd7, Nedd9 and Vim and the VEGF-α genes in 4T1 cells and mice tumor and lung tissues [94,95].

In a complementary piece of research, our group investigated the anti-metastatic efficiency of the combination of Crocin and Crocetin, another major carotenoid of saffron, on the cell and mice models of TNBC. It was shown that the combination therapy of 4T1 cells reduced proliferation, migration, invasion and adhesion to ECM, even more effectively than Crocin and/or Crocetin. The xenograft mice assays confirmed these outcomes, as the combination-treated mice possessed less metastatic deposits in the liver and lung tissues. The Western blot and real-time PCR findings exhibited that the combination therapy reduced the expressions of Fzd7, Nedd9, VEGF-α, Vim and Mmp9 and upregulated E-Cad in 4T1 cells and liver and tumor tissues, thus preventing Wnt/β-catenin signaling and averting metastasis in TNBC [96].

**Figure 1:**
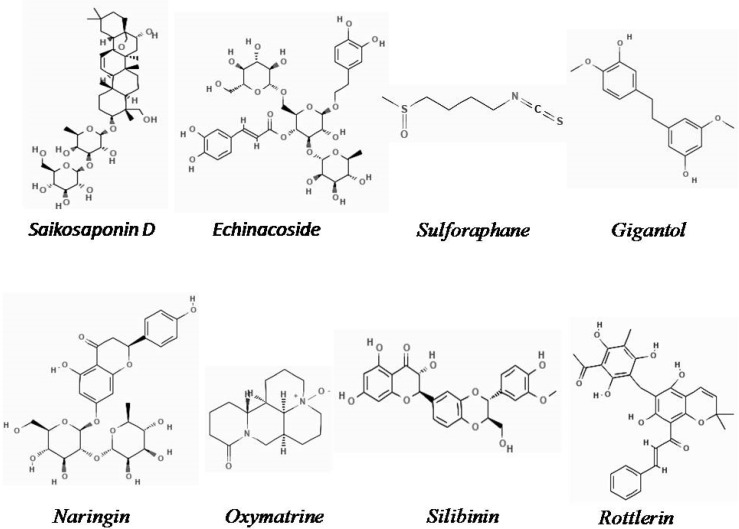

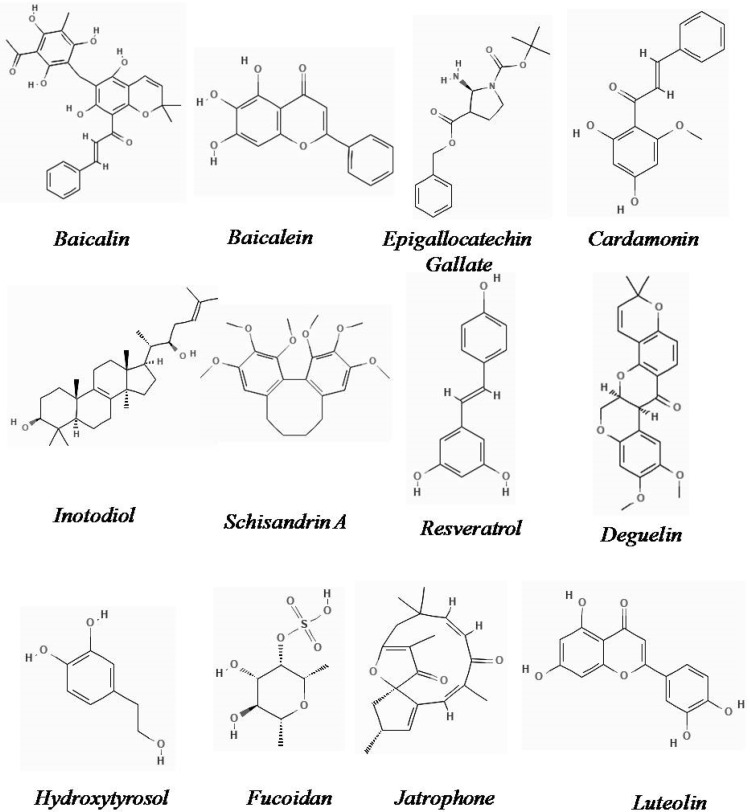

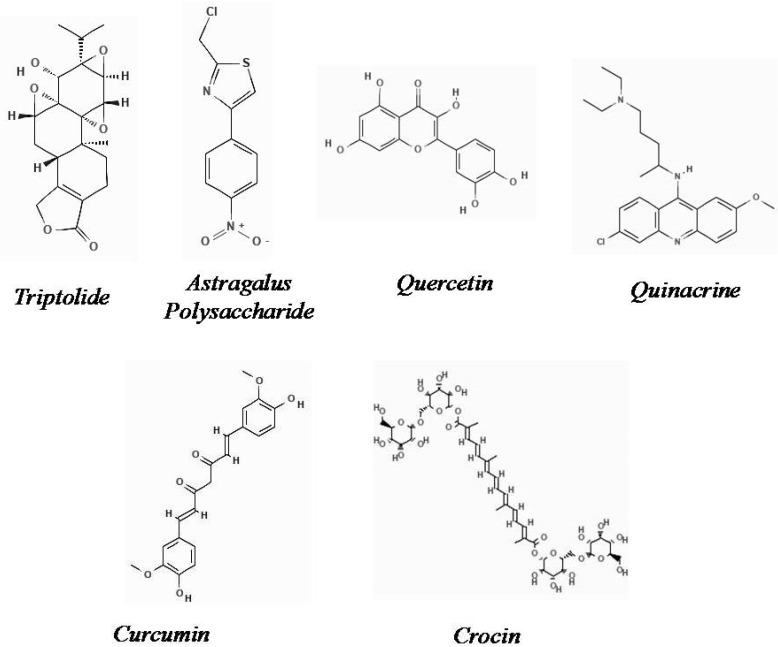
Chemical structure of natural derivatives combating TNBC through interfering with the Wnt/β-catenin pathway (Pubchem).

The chemical structures of the natural derivatives combating TNBC through interfering with the Wnt/β-catenin pathway are demonstrated in Figure 1.

## 3. Natural Pill or Extracts Targeting TNBC via Impeding the Wnt/β-Catenin Pathway

### 3.1. Liuwei Dihuang Pill

The Liuwei Dihuang pill (LWDHP) is a classic Chinese herbal medicine containing six herbs: *Rehmannia glutinosa* (Gaetn.) Libosch. ex Fisch. et Mey (Shudi Huang); *Dioscorea opposita* Thunb (Shan Yao); *Cornus officinalis Sieb*. et Zucc (Shanzhu Yu); *Poriacocos* (Schw.) Wolf (Fu Ling); *Alisma orientalis* (Sam) Juzep (ZeXie) and *Paeonia suffruticosa* Andr (Mudan Pi) [97]. It has been prescribed for the prevention and treatment of breast cancer, particularly TNBC [98,99]. Lixiang et al.’s research showed that LWDHP therapy restricted the tumor volume and elevated the survival rate of spontaneous breast carcinoma mice [100]. Moreover, in 2019, they confirmed that the LWDHP reduced the cancer tissue size and weight, increased the cancer inhibitory rate, prolonged the survival time and prohibited lung or liver metastasis in TNBC-bearing mice compared to the control mice. Their results suggested that the LWDHP modulates the deregulation of the Wnt signaling pathway since, β-catenin, CyclinD1, TCF-1 and VEGF expressions decreased. However, high doses of LWDHP augmented the expression of Axin-2. In addition, this study confirmed that LWDHP disrupted the β-catenin/TCF-1 interaction in the nuclei. It should be noted that the activation of the β-catenin/TCF-1 complexes in Wnt signaling promoted the transcription of the downstream genes e.g., VEGF and CyclinD1, and elevated cell proliferation [101].

### 3.2. Syzygium guineense

*Syzygium guineense* Wall., a member of the *Myrtaceae* family, is a widespread plant native to the regions of Australia, Asia and Africa. It has been prescribed as a medicinal plant due to its anti-tumor activity against TNBC cells [102,103]. Koval et al. reported that Wnt3a is strikingly overexpressed in TNBC. They demonstrated that the *S. guineense* extract therapy of malignant cells lead to an inhibition of cell proliferation through blocking the Wnt3a-induced β-catenin stabilization and suppression of the Wnt-dependent transcription. It is also noteworthy that phyto-analytical studies have introduced tannins, a group of polyphenols, as an effective inhibitor of Wnt3a-stimulated signaling and TNBC cell proliferation [103].

### 3.3. Ganoderma lucidum

*Ganoderma lucidum* (Reishi or Lingzhi), the most common species in the genus *Ganoderma*, is a famous Chinese traditional medicinal plant, which has found novel applications as a cancer adjunct-therapy agent. The phytochemical research elucidated that the antitumor property of Ganoderma extract is due to its constituent triterpenes [104]. The studies on SUM-149 and mice models have provided evidence of Reishi’s therapeutic potential for breast cancer treatment [105]. Zhang et al., in 2017, elucidated its anti-cancer molecular mechanism, indicating that Reishi diminished cell viability and migration via suppressing the phosphorylation of the Wnt co-receptor LRP6, leading to blocking the Wnt/β-catenin signaling pathway in human and mouse breast cancer cell lines [106].

## 4. Conclusions

Natural compounds are proposed to compensate for the undesirable effects of chemotherapeutic drugs. Frequently, the bio-accessibility and bioavailability of natural agents, and their nontoxicity on normal cells and anti-tumor/anti-metastatic potentials nominates natural agents as propitious, multipotent chemopreventive and chemotherapeutic remedies. Various surveys on cells and animal models of TNBC revealed the suppressive efficacy of herbal medicine extracts and bioactive compounds including: flavonoids; terpenes; carotenoids; alkaloids; retinoids and lignans on various molecules of the Wnt/β-catenin signaling pathway, WNT, FZD, LRP, GSK3β, APC, Dsh, β-catenin and TCF/LEF. These phytotherapy agents exerted anti-proliferation, proapoptotic, anti-migration and anti-invasion effects and the inhibition of BCSC self-renewal via modulation of the Wnt downstream genes (c-Myc, Cyclin D1, Survivin, Axin2, and EMT-related genes). The administration of a number of these herbal medications simultaneously repressed Wnt and mTORC1, ER stress and SATB1 signaling (Table 1). The co-administration of natural compounds and common chemotherapy agents ameliorates the function of chemotherapeutics. However, it should be considered that the combination therapy may have adverse consequences through pharmacodynamic and pharmacokinetic herb–chemotherapeutic interactions. Therefore, an outlook and prospect for the future would be the evaluation of natural bioactive compounds in clinical trials. In the absence of efficient approved targeted therapies, these findings open a new avenue on the role of herbal extracts and derivatives as an auspicious medication for the treatment of TNBC sufferers.

## Figures and Tables

**Table 1:** Anticancer potentials of active phyto-therapeutic ingredients against triple negative breast cancer.

Active Pharmaceutical Ingredient	Plant	Model of Study	Dose	Targeted Genes	Impact on TNBC	Reference
Saikosaponin D	*Radix Bupleuri*	HCC1937 MDA-MB-468 MDA-MB-231	10, 15 and 20 µM	*β-Catenin* *↓* *Cyclin D1* *↓* *c-Myc↓*	Proliferation↓ Apoptosis↑	[23]
Echinacoside	*Cistanche* and *Echinacea*	MDA-MB-468 MDA-MB-231	25–100µM	*p-LRP6↓* *total LRP6↓* *p-Dvl2↓* *active β-Catenin↓* *total β-Catenin↓* *CD44↓* *LEF1↓* *Cyclin D↓*	Proliferation↓ Migration↓ Invasion↓ Tumor sizes↓ Tumor weights↓	[25]
		Nude mice	10 mg/kg			
Sulforaphane	*Brassica*	SUM159	0.5, 1 and 5 µM	*β-Catenin↓* *Cyclin D1↓*	Proliferation↓ Apoptosis↑ Mammosphere formation↓ BCSC activity↓ Tumor sizes↓	[27]
		NOD/SCID mice	50 mg/kg			
Gigantol	*Orchidaceae*	MDA-MB-231 MDA-MB-468	0–100 µM	*p-LRP6*,*↓* *total LRP6↓* *Cytosolic β-catenin↓* *Axin2↓* *Survivin↓*	Proliferation↓ Migration↓	[29]
Naringin	*Drynaria fortunei* *Citrus aurantium Citrus medicaand* citrus fruit	MDA-MB-231 MDA-MB-468 BT-549	0–200 µM	*Survivin↓* *P21↓* *β-Catenin ↓* *Cyclin E↓* *Rb ↓* *p-Rb↓*	Proliferation↓ Apoptosis↑ Arrest cell cycle in G1 Tumor sizes↓ Tumor weights↓	[31]
		SCID hairless mice	100 mg/kg			
Oxymatrine	*Sophora* *Flavescens* Ait	MDA-MB-231 MDA-MB-468	0, 1, 2 and 4 mM Bevacizumab 200 nM	*N-Cad↓* *Vim↓* *ZEB↓* *Snail↓* *Slug↓* *E-Cad↑* *β-Catenin↓* *c-Myc↓* *Cyclin D1↓* *CD44↓* *VegfA↓*	Migration↓ Invasion↓ EMT↓ BCSC self-renewal↓ Tumor size↓ Angiogenesis↓	[34]
		BALB/c nude mice	25 mg/kg + 5 mg/kg Bevacizumab			
Silibinin	*Silybum marianum*. Gaertn	MDA-MB-231	0–200 µM	*LRP6↓* *p-LRP6↓* *Axin2↓*	Proliferation↓	[38]
Rottlerin	*Mallotus* *philippinensis*	MDA-MB-231	0.1–31.6 μM	*cytosolic* *β-catenin↓* *total* *β-Catenin↓* *LRP6↓* *p--LRP6↓* *Axin2↓* *P70-S6K↓* *p-P70-S6K↓* *S6↓* *p-S6↓* *CyclinD1↓* *Survivin↓*	Proliferation↓	[41]
Baicalin	*Scutellaria* *baicalensis* Georgi	MDA-MB-231 4T1	10, 30 and 100 μM	*β-Catenin↓* *E-Cad↑* *Claudin↑* *N-Cad↓* *VIM↓* *Snail↓* *Slug↓*	Proliferation↓ Migration↓ Invasion↓ EMT↓ Metastatic colonies in liver and lung↓	[44]
		BALB/c mice	100 mg/kg			
Baicalein	*Scutellaria* *baicalensis* Georgi	MDA-MB-231	10, 20, and 40 μM/L	*E-Cad↑* *Vim↓* *Snail↓* *Wnt1↓* *β-Catenin↓* *SATB1↓* *Cyclin D1↓* *Axin2↓*	Proliferation↓ Migration↓ Invasion↓ EMT↓ Metastatic colonies in liver and lung↓	[46]
		BALB/c nude mice	50 or 100 mg/kg			
Epigallocatechin gallate	Green tea	MDA-MB-231	25, 50, 75, 100 and 200 μM	*β-Catenin* *↓* *p-AKT* *↓* *Cyclin D1* *↓*	Proliferation↓	[48]
		MDA-MB-231	25–100 μM	*HBP1↓* *β-Catenin↓* *c-Myc↓*	Proliferation↓ Migration↓ Invasion↓	[50]
Cardamonin	*Zingiberaceae*	MDA-MB-231 BT-549	0–100 μM	*Bax↑* *Bcl-2↓* *Cyt-C↑* *Caspase-3↑* *PARP↑* *β-Catenin↓* *E Cad↑* *N-Cad↓* *Vim↓* *Slug↓* *Snail↓* *Cyclin D1↓* *c-Myc↓* *Vegf↓* *CDK4↓*	Proliferation↓ Migration↓ Invasion↓ EMT↓ Apoptosis↑ Arrest cell cycle in G2/M Tumor size↓	[53]
		BALB/c mice	2.5 and 5 mg/kg			
Inotodiol	*Inonotus obliquus*	Sprague-Dawley rats	10 mg/kg	*PCNA↓* *β-Catenin↓* *c-Myc↓* *Cyclin D1↓* *Caspase-3↑* *PARP↑*	Proliferation↓ Apoptosis↑ Body weight↑ Antioxidant enzyme activities↑ Abnormal histological signs of pancreas↓ Glucose↓ Cholesterol↓ Triglyceride↓ HDL↓	[56]
Schisandrin	*Schisandra* *chinensis* Baill	MDA-MB-231 BT-549	25,50 and 100 µM	*ATF4↑* *CHOP ↑* *p-Elf2α↑* *β-Catenin* *↓* *p-GSK3β↓* *Bax↑* *P53↑* *Bcl2↓*	Proliferation↓ Arrest cell cycle in G1 Apoptosis↑ ER stress↑ Tumor size↓	[60]
		BALB/c mice	25 mg/kg			
Resveratrol	Grapes, Berries and Peanuts	SUM159	10, 20 and 40µM	*LC3-II↑* *Beclin1↑* *Atg 7↑* *β-Catenin* *↓* *Cyclin D1* *↓*	Proliferation↓ BCSC activity↓ BCSC self-renewal↓ Autophagy↑ Tumor size↓	[64]
		NOD/SCID mice	100 mg/kg			
Deguelin	*Mundulea sericea*	MDA-MB-231	0.1–10 μM/L	*WIF-1* *↑* *DDK4* *↑* *CDH3* *↑* *CDH7* *↑* *CDH9* *↑* *Wnt14* *↓* *Wnt 2B* *↓* *Wnt 3* *↓* *Snail* *↓* *p-GSK-3β↓* *β-Catenin* *↓* *Cyclin D1* *↓*	Proliferation↓ Apoptosis↑ Arrest cell cycle in S	[67]
Hydroxytyrosol	Olive oil	SUM159PT BT549 MDA-MB-231 Hs578T	0–100 μM	*Zeb↓* *Slug↓* *Vim↓* *Zo-1↑* *p-LRP6↓* *LRP6↓* *β-Catenin↓* *Cyclin D1↓*	BCSC self-renewal↓ Migration↓ Invasion↓ EMT↓ BCSC activity↓	[69]
Fucoidan	Brown seaweed and Marine invertebrates	4T1	50, 100 and 200 µg/mL	*β-Catenin* *↓* *c-Myc* *↓* *Cyclin D1↓* *Survivin* *↓* *TCF/LEF↓*	Proliferation↓ Apoptosis↑ Arrest cell cycle in G1 Tumor size↓ Tumor weight↓	[72]
		BALB/c mice	5, 10 mg/kg			
Jatrophone	Euphorbiaceae	MDA-MB-231 MDA-MB-157 HCC38 MDA-MB-468 Patient-derived xenograft	100 nM- 30 μM	*BIRC5* *↓* *Axin2* *↓* *HMGA2* *↓* *Myc* *↓* *PCNA* *↓* *CCND1* *↓* *Cyclin D1* *↓* *β-Catenin↓* *Slug↓* *Fibronectin↓* *Vim↓*	Proliferation↓ Apoptosis↑ Arrest cell cycle in S-phase EMT↓ Migration↓	[75]
Luteolin	Celery Sweet bell peppers, Carrots Broccoli Onion leaves Parsley	MDA-MB-231 BT5–49	10, 30 and 100 μM	*N-Cad↓* *Vim↓* *Snail↓* *Slug↓* *E-Cad↑* *Claudin↑* *β-Catenin* *↓*	Migration↓ Invasion↓ EMT↓ Metastatic colonies↓	[77]
		Nude mice	100 mg/kg			
Triptolide	*Tripterygium* *wilfordii Hook F*	MDA-MB-231	10, 25 and 50 nM	*β-Catenin↓*	Proliferation↓ Apoptosis↑	[79]
Astragalus polysaccharide	*Astragalus membranaceus*	MDA-MB-231	25, 50, 100, 200, 400, 800 and 1600 μg/mL	*Vim↓* *Snail↓* *E-Cad↑* *β-Catenin↓* *c-Myc↓* *Cycline D1↓*	Proliferation↓ Migration↓ Invasion↓ EMT↓	[81]
Quercetin	Fruits, Vegetables, Seeds, Nuts, Green tea, and Red wine	MDA-MB-231 MDA-MB-468	10 and 50 μM	*Vim↓* *E-Cad↑* *Cyclin D1* *↓* *c-Myc* *↓* *p-AKT* *↓*	Proliferation↓ Survival rate↓ Migration↓ Invasion↓ Reshape mesenchymal to epithelial shape	[83]
Quinacrine	Cinchona tree	MDA-MB-231	5, 10, 15 and 20 μM	*β-Catenin* *↓* *Cyclin D1* *↓* *APC↑*	Proliferation↓ Survival rate↓ Apoptosis↑ DNA damage↑ Topoisomerase activity↓	[86,87]
Quinacrine + Lycopen	Cinchona tree Tomato		5 μM + 2, 4, 8, 10, 12, 14 and 16 μM	*β-Catenin* *↓* *Cyclin D1* *↓* *APC↑*	Proliferation↓ Survival ↓	
Curcumin	*Curcuma longa*	MDA-MB-231	20 μM	*Dsh↓* *β-Catenin↓*, *Cyclin D1↓* *Slug↓*	Proliferation↓ Apoptosis↑ Arrest cell cycle in G2/M	[89]
		SUM159	10, 20 and 40 μM	*CD44↓* *ALDH1A1↓* *Nanog↓* *OCT4↓* *PCNA↓* *Cyclin D1↓* *Bcl2↓* *Bax↑*. *Caspase 8↑* *Caspase9↑* *cleaved* *Caspase 3↑* *p-GSK3β↓* *β-Catenin↓* *c-Myc↓*	Proliferation↓ Apoptosis↑ Mammosphere formation↓ BCSC activity↓	[90]
Crocin	*Crocus sativus*	4T1	2.5 and 3 mM	*FzD7↓* *Nedd9↓* *Vegf-α↓* *Mmp9↓* *Vim↓* *E-Cad↑*	Proliferation↓ Invasion↓ Migration↓ Cell–ECM adhesion↓ Tumor size↓ Metastatic colonies↓	[94]
		BALB/c mice	200 mg/kg			[95]
Crocin + Crocetin	*Crocus sativus*	4T1	Crocin 2.5 mM + Crocetin 0.05 mM & Crocetin 0.1 mM + Crocin 2 mM	*Fzd7↓* *Nedd 9↓* *Vegf-α↓* *Mmp9↓* *Vim↓*	Proliferation↓ Invasion↓ Migration↓ Tumor size↓ Cell–ECM adhesion↓ Metastatic colonies↓	[96]
		BALB/c mice	Crocin 200 mg/kg + Crocetin 5 mg/kg			
Liuwei Dihuang pill	*Rehmannia glutinosa* *Dioscorea opposita* *Cornus officinalis* *Poria cocos* *Alisma orientalis* *Paeonia suffruticosa*	Kunming mice	2.3, 4.6 and 9.2 g/kg	*β-Catenin↓* *Cyclin D1↓* *TCF-1↓* *Vegf↓*	Tumor sizes↓ Tumor weights↓ Survival time↑ Metastatic colonies in liver and lung↓	[101]
Tannins	*Syzygium* *guineense* Wall	BT-20 HCC38 MDA-MB-231, HCC1806 HCC1395 MDA-MB-468	0–100 μg/mL	*Wnt3a↓* *β-Catenin↓* *LRP6↓*	Proliferation↓	[103]
Ganoderma Lucidum	_	MDA-MB-231 4T1	0–200 µg/mL	*β-Catenin↓* *p-LRP6↓* *p-Dvl2↓* *Axin2↓*	Proliferation ↓ Migration↓	[106]

## Data Availability

Not applicable.
